# Influence of Freeze–Thaw Cycles and Binder Dosage on the Engineering Properties of Compound Solidified/Stabilized Lead-Contaminated Soils

**DOI:** 10.3390/ijerph17031077

**Published:** 2020-02-08

**Authors:** Zhongping Yang, Yao Wang, Denghua Li, Xuyong Li, Xinrong Liu

**Affiliations:** 1 College of Civil Engineering, Chongqing University, Chongqing 400045, China; 2 Key Laboratory of New Technology for Construction of Cities in Mountain Area (Chongqing University), Ministry of Education, Chongqing 400045, China; 3 National Joint Engineering Research Center for Prevention and Control of Environmental Geological Hazards in the TGR Area Chongqing University, Chongqing 400045, China

**Keywords:** solidification/stabilization, freeze–thaw cycles, dosage, lead-contaminated, excessive cementation, engineering characteristics, deterioration

## Abstract

The solidification/stabilization (S/S) method is the usual technique for the remediation of soils polluted by heavy metal in recent years. However, freeze–thaw cycles, an important physical process producing weathering of materials, will affect the long-term stability of engineering characteristics in solidified contaminated soil. In addition, it is still questionable whether using large dosages of binders can enhance the engineering properties of solidified/stabilized contaminated soils. In this study, the three most commonly used binders (i.e., cement, quicklime, and fly ash), alone and mixed in different ratios, were thus added to lead-contaminated soil in various dosages, making a series of cured lead-contaminated soils with different dosages of binders. Afterward, unconfined compression strength tests, direct shear tests, and permeability tests were employed on the resulting samples to find the unconfined compressive strength (UCS), secant modulus ($E_{50}$), internal friction angle (φ), cohesion (c), and permeability coefficient (k) of each solidified/stabilized lead-contaminated soil after 0, 3, 7, and 14 days of freeze–thaw cycles. This procedure was aimed at evaluating the influence of freeze–thaw cycle and binder dosage on engineering properties of solidified/stabilized lead-contaminated soils. Results of our experiments showed that cement/quicklime/fly ash could remediate lead-contaminated soils. However, it did not mean that the more the dosage of binder, the better the curing effect. There was a critical dosage. Excessive cementation of contaminated soils caused by too much binder would result in loss of strength and an increase in permeability. Furthermore, it was found that UCS, ${\;E}_{50}$, φ, c, and k values generally decreased with the increase in freeze–thaw cycle time—a deterioration effect on the engineering characteristics of solidified lead-contaminated soils. Avoiding excessive cementation, 2.5% cement or quicklime was favorable for the value of $E_{50}$ while a 2.5% fly ash additive was beneficial for the k value. It is also suggested that if the freeze–thaw cycle continues beyond the period supported by excessive cementation, such a cycle will rapidly destroy the original structure of the soil and create large cracks, leading to an increase in permeability. The results also showed that the contaminated soils with a larger dosage of binders exhibited more significant deterioration during freeze–thaw cycles.

## 1. Introduction

Over the past 30 decades, the rapid development of industry and agriculture have led to soils contaminated by heavy metals worldwide [1,2,3,4,5,6]. An investigation performed by Wei and Yang [7] found that approximately 65% of cities had high or extremely high levels of contamination with heavy metals in urban soils. Likewise, Fan et al. [8] reported that in some contaminated sites, the heavy metal content reached as high as 50~7000 mg·kg^−1^, in particular, the lead content in some sites was as high as 424,000 mg·kg^−1^, and the strontium content was as high as 1,700,000 mg·kg^−1^. These reports demonstrated that heavy metal pollution has become the key factor in soil pollution in China.

If heavy metal contaminated soils remain untreated over time, heavy metal contaminants likely will percolate down to groundwater or discharge to rivers [9]. As such, heavy metals could enter health systems through the food chain [10] due to accumulation in plants, a process relatively unaffected by beneficial microorganism action [11], which might result in adverse effects on the environment [12] and human health [13,14]. Such contamination can also affect the basic physical properties and engineering mechanical properties of the foundation soil, such as the void ratio, compressibility, shear strength, and bearing capacity, etc. [10,15,16,17,18]. Therefore, the effective treatment of heavy metal contaminated soils is urgently needed in terms of protecting the environment, improving the engineering properties of the foundation soil, and realizing the reuse of contaminated industrial sites [19].

Among the existing remedial measures for heavy metal contaminated soils, the solidification/stabilization (S/S) technique stands out as being low-risk, relatively cost-effective, simple to operate, and widely applicable to engineering, as well as increasing comprehensive soil strength, and offering good resistance to biodegradation [20,21].

S/S is a remediation technology that reduces the toxicity of pollutants, by using physical or chemical methods to either immobilize harmful pollutants contained in the soil or to convert such pollutants into chemically inactive forms, preventing their migration and diffusion into the environment [22,23]. Solidification is defined as a process in which materials are added to waste to come into stable solids, while stabilization, on the other hand, refers to a chemical remediation process that uses chemical reaction to reduce the solubility and migration of pollutants in soils or converts them into chemically inert substances, reducing the hazards level of such wastes [24,25].

Du et al. [26] confirmed that the main mechanisms of S/S technology are the chemical interaction of hydration reaction products (such as calcium silicate hydrate, CSH) with pollutants or their adsorption, which physically retains contaminants through the surfaces of various hydrates. These products are closely related to the binders [20,27], mainly involving cement, slag, fly ash, quicklime, modified clay (such as organic clay), lime, and some waste [27,28,29]. For example, Wang et al. [30] found that when the dosage of magnesium phosphate cement added to the soils increased from 30% to 70%, the leaching amount of lead seen in subsequent leaching was significantly reduced. Wang et al. [31] further proved that an increase in the dosage of binders caused the integrity of a GMC (GGBS-MgO-CaO) mixture to be improved after several drying and wetting cycles. However, it remains inconclusive whether the engineering characteristics of the solidified contaminated soil will continue to improve if the dosage of the binder increases further.

On the other hand, researches, done by Aldaood et al. [32], Du et al. [33], Li et al. [34], Zaimoglu [35], and Xu et al. [36], showed that extreme environmental changes, such as freeze–thaw cycles, high-salt groundwater, acid rain infiltration, or dry-wet alternation, could result in changes in the properties of solidified contaminated soils and even cause the S/S to fail. Regions in China subjected to freezing are widely distributed: For example, permafrost regions accounts for approximately 21.5% of the total land area, seasonal frozen soil regions about 53.5%, transient frozen soil regions approximately 23.9%, and frozen soil-free regions only 1.1% according to the report by Xia [37]. Apparently, freeze–thaw alternation is an important abiotic force that affects the soil environment in China.

Under the action of freeze–thaw cycles, changes in liquid water cause changes in the three-phase composition ratio of the soil as well as changes in the size and stability of soil aggregates, leading to increases in permeability and decreases in soil stability. In addition, freezing and thawing causes aggregates to release a large amount of inorganic substances, which enhances the organic mineralization of soil, adsorption, and desorption of organics. The freezing and thawing also affect morphological transformation, microbial activity, and the free energy stored in the soil. These changes in the physical and chemical properties of the soil inevitably affect the occurrence, form, and migration of heavy metal pollutants, adsorbed within the soil and the particles of the solidifying agent or colloidal surface [38,39,40], leading to changes in the structure and the physical and mechanical properties of solidified polluted soils. This causes the engineering mechanical properties of contaminated soil in such regions to differ from those seen in non-freeze–thaw regions and thus affects the reconstruction of sites abandoned due to pollution [18,41]. For instance, Roustaei et al. [42] reported that the undrained triaxial compressive strength of both unreinforced and reinforced soil decreased as the number of freeze–thaw cycles increased. According to Wang et al. [43], the dynamic modulus decreased significantly, and the damping ratio increased with an increase in the number of freeze–thaw cycles. However, there are few studies on how the secant modulus ($E_{50}$) and shear index (φ and c) of solidified contaminated soil change when this is subjected to freeze–thaw cycles.

Based on the above evidences, taking the cement/quicklime/fly ash commonly used in soil S/S into consideration [44], the aim of the present study was to systematically investigate the effects of the freeze–thaw cycle and binder dosage on the engineering properties of solidified/stabilized lead-contaminated soils remediated with cement/quicklime/fly ash alone or some mixture of these additives. For comparison purposes, the engineering properties of lead-contaminated soils without solidified/stabilized were also tested. A series of experiments, including unconfined compression strength (UCS) tests, direct shear tests, and permeability tests, were performed on soil samples subjected to freeze–thaw cycles for 0, 3, 7, or 14 days to investigate the effect of these freeze–thaw cycles and various binder dosages (0%, 2.5%, and 5%) on the values of UCS secant modulus ($E_{50}$), internal friction angle (φ), cohesion (c), and permeability coefficient (k) in compound solidified/stabilized lead-contaminated soils. And combining with the direct trend chart of influencing factors, the dosage of binder in the solidified lead-contaminated soil under the freeze–thaw cycle, which could make engineering properties (UCS, $E_{50}$, φ, c, and k) better, was further studied.

## 2. Materials and Methods

### 2.1. Materials

**Figure 1:**
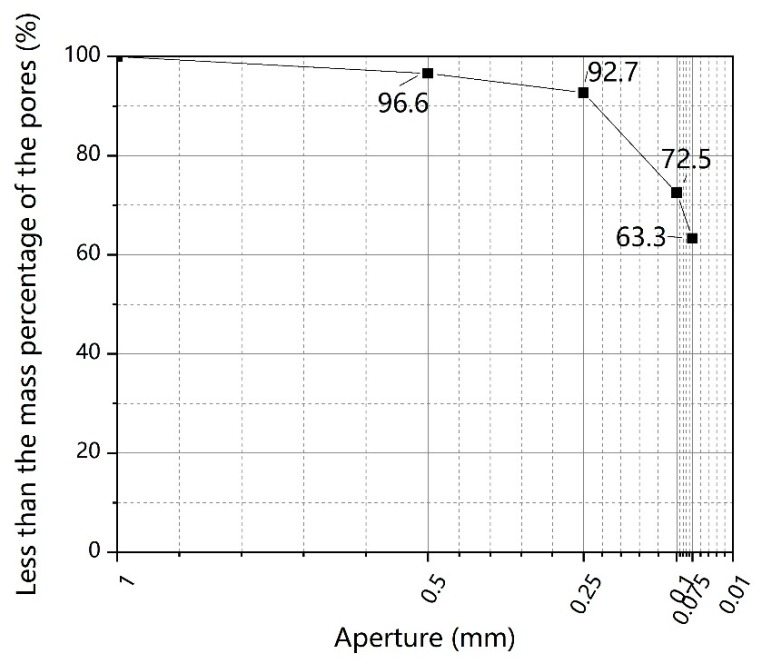
Gradation curve of undisturbed soil.

Undisturbed soils used in this work were taken from an industrial site in Chongqing, China, being reddish-brown in color. The collected soil samples were air-dried in the laboratory for about two weeks, and afterward, they were sieved through a 16-mesh polyethylene screen (<1 mm) to remove stones, branches, coarse materials, and other debris. Subsequently, the sieved soil samples were subjected to conventional soil tests in accordance with the Standards for Geotechnical Test Methods (GB/T 50123-1999). The physical property parameters of the soil samples are shown in Table 1. The soils passed through a 1 mm sieve were subjected to sieving treatment with an electric sifter to obtain a particle gradation curve (as shown in Figure 1. According to the Engineering Classification Standard for Soil (GB/T 50145-2007), the soils were classified as fine-grained soil containing coarse grain, and with a liquid-low limit. The main chemical component of the tested soils is ${\mathrm{SiO}}_{2}$, determined by the X-ray fluorescence (XRF) technique (as shown in Table 2).

In this experiment, ${\mathrm{Pb}}^{2+}$ was selected as the contaminating ion. In view of the high solubility of nitrate (high cation mobility) and its minimal interference to the S/S process [45,46], analytical-grade $\mathrm{Pb}{\left({\mathrm{NO}}_{3}\right)}_{2}$ was used as a heavy metal contaminant. The quality indicators for analytical-grade $\mathrm{Pb}{\left({\mathrm{NO}}_{3}\right)}_{2}$, as purchased from Sinopharm Group Reagent Co., Ltd., are shown in Table 3.

Three binders (i.e., cement, quicklime, and fly ash) were selected to remediate lead-contaminated soils in the current research. The cement selected was ordinary Portland cement (OPC325); the main component of quicklime is calcium oxide (here of an analytical grade); while the fly ash was obtained from the Chongqing Power Plant (secondary). As the total ${\mathrm{SiO}}_{2}$, ${\mathrm{Al}}_{2}O_{3},$ and ${\mathrm{Fe}}_{2}O_{3}$ content in the fly ash exceeded 70%, the fly ash used could be classified as Class F. All the three binders were passed through a 0.075 mm sieve to develop uniformity and the underlying chemical compositions of the cement, quicklime, and fly ash were obtained by X-ray fluorescence (XRF) testing, as shown in Table 4.

### 2.2. Specimen Preparation

In this research, heavy metal pollutants of predetermined weights (mass ratio of ${\mathrm{Pb}}^{2+}$ to dry soils or a ${\mathrm{Pb}}^{2+}$ concentration value was 10,000 mg/kg) were dissolved in deionized water. Then these solutions were thoroughly blended with the dry soils using a magnetic stirrer for about 10 min, and each solution-soil mixture was sealed in a humidity box (temperature of 22 °C and relative humidity of 95%) for three months to obtain man-made lead-contaminated soils.

To investigate the effect of binder dosage on engineering characteristics, some of the polluted soils made in this way were divided into nine portions. One of each of nine kinds of different binders, made of cement (0%, 2.5%, or 5%), quicklime (0%, 2.5%, or 5%) and fly ash (0%, 2.5%, or 5%) alone or mixed, were respectively added into these, and the mixtures of contaminated soils with binders were all mechanically stirred for more than 10 min with a magnetic stirrer. For comparison, specimens without any binder added were also prepared.

For the sake of achieving optimal moisture content (OMC), as shown in Table 1, a certain amount of deionized water was added into the soil specimens. Under this OMC, UCS, and other mechanical properties of the soils were at their best conditions [47].

Mass of prepared mixtures required for each test, calculated based on this OMC, 95% of the maximum dry density and specimen size, was weighed and transferred into a Φ39.1 × 80 mm cylindrical mold. Next, the mold was placed onto a vibrating stand with the frequency and amplitude of 48 Hz and 0.5 mm to obtain specimens of a diameter of 39.1mm, a height of 80mm for the UCS tests and permeability coefficient tests. Similarly, the weighed mixtures were transferred into a sample container with a ring knife and were statically compacted to acquired direct shear tests sample with a diameter of 61.8 mm, and a height of 20 mm. After extracting from the molds by using a hydraulic jack, all specimens were sealed with a plastic film to prevent loss of moisture before being placed into a standard curing room (temperature of 22 °C and relative humidity of 95%) for 56 days.

The testing procedure of compound lead-contaminated soils treated with binder mixtures of various dosages (0%, 2.5%, or 5%) is shown in Table 5, which also lists the concentration of each binder in the various formulations. Here, “Pbi” denotes a specimen with a Pb concentration of i% based on its oven-dried soil weight. Analogously, “Cj”, “Sk”, and “Fm” denote samples with cement concentrations of j%, quicklime concentrations of k%, or fly ash concentrations of m%, respectively.

### 2.3. Testing Methods

#### 2.3.1. Freeze–Thaw Cycle Test

**Figure 2:**
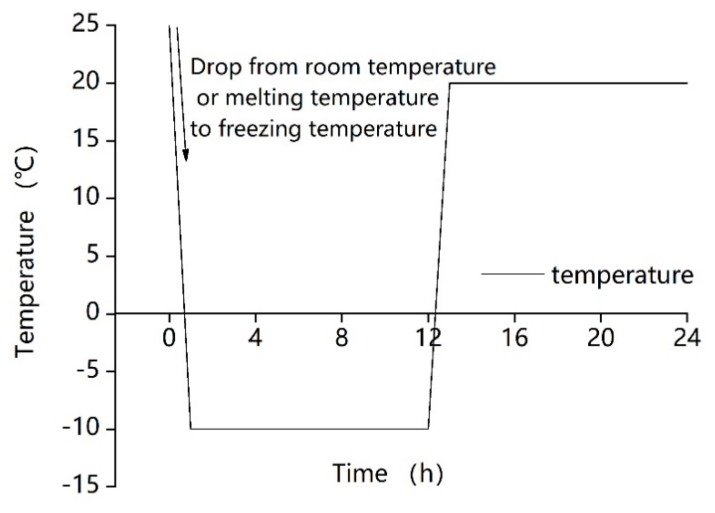
Temperature curve of the one-day freeze–thaw cycle.

The cured samples were put into an alternating high- and low-temperature test chamber (TC401, Chongqing Taisite Test Instrument Co. Ltd.; temperature range: −40 °C~100 °C) at Chongqing University for the freeze–thaw cycle tests. The freeze–thaw cycles were set to 0 days, 3 days, 7 days, and 14 days, respectively. The one-day freeze–thaw cycle is shown in Figure 2. And as shown, after the sample was placed into the freeze–thaw cycle test chamber, the temperature was dropped from room temperature to −10 °C in one hour. After maintaining this temperature for 11 h, it was then increased to 20 °C within one hour, and this temperature was then maintained for a further 11 h.

#### 2.3.2. Unconfined Compression Strength (UCS) Test

Following the Chinese standard for soil test methods (GB/T 50123-2019), the unconfined compressive strength (UCS) was tested using the YYW-2 strain-controlled unconfined controller (Manufacturer: Nanjing Ningxi Soil Instrument Co., Ltd., Nanjing, China). The test was carried out by the displacement control method (1 mm/min) loading, which defined the secant modulus $E_{50}$ as the ratio of stress and strain when the on the sample is half of its peak stress.

The axial strain rate should be in the range of 1% to 3% strain per minute. The lifting device for testing was raised by turning the handle. When the axial strain was less than 3%, the data were read at every 0.5% of strain (0.4 mm); when the axial strain was equal to or greater than 3%, the data were read at every 1% of strain (0.8 mm). Each test was completed in under 10 min. When the reading of dynamometer peaked, the test ceased after continuing the strain of 3% to 5%; when there was no peak in the reading, the test continued until the strain reached 20%.

#### 2.3.3. Direct Shear Test

The shear strength was tested with a ZJ-2 strain-controlled direct shear instrument (Manufacturer: Nanjing soil instrument factory Co., Ltd., Nanjing, China). The shear rate was 0.8 mm/min. When the sample showed a displacement of 0.2 to 0.4 mm, the measurement of the proving ring and the shear displacement of the box were recorded. When the peak value of the proving ring dial gage was reached, the instrument continued to shear until the displacement reached 4 mm, at which point the damage value was recorded. Where the machine stopped during the shearing process, the damage value was also recorded. If the maximum value of the dial indicator reading was reached during the shearing process, the instrument continued to apply shear stress until the displacement reached 6 mm [48,49].

#### 2.3.4. Permeability Test

**Figure 3:**
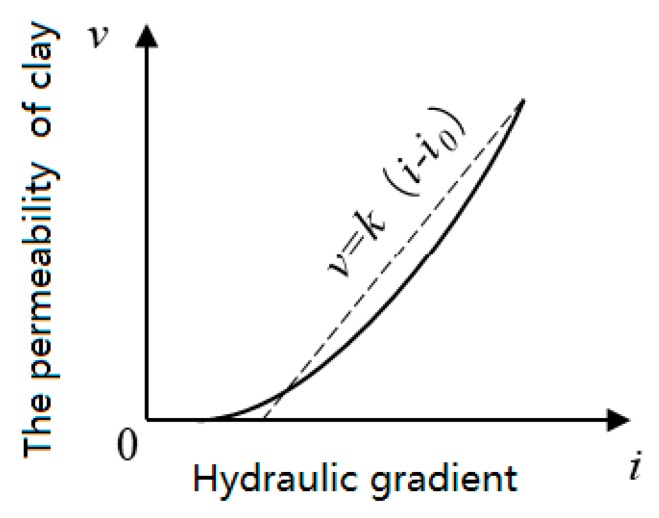
Relationship between the permeability rate of clay and the hydraulic gradient.

The permeability coefficient (k) was tested using an automatic environmental, geotechnical penetration meter (GDSPERM, GDS Instruments Ltd., Hook, UK). A specimen that had been subjected to vacuum saturation for 24 h was loaded into a pressure chamber, and the confining pressure, back pressure, and reaction force difference were set to determine its permeability coefficient. As the permeability coefficient of clay increases with the increase in hydraulic slope drop, its value approaches a constant [49] when the hydraulic slope was large. After testing, it was found that when the hydraulic head pressure exceeded 50 kPa, the instrument pressure tended to become unstable, and the samples were damaged internally. Therefore, the confining pressure was set at 100 kPa, and the osmotic pressure changed from 10 kPa to 20 kPa and then to 30 kPa, 40 kPa, and 50 kPa before dropping to 40 kPa and thence, to 30 kPa, 20 kPa, and finally, 10 kPa. The relationship between the permeability rate of the clay soil and the hydraulic gradient (shown as in Figure 3) [49] was thus utilized to obtain a stable permeability coefficient for the sample.

## 3. Results

### 3.1. Influence of Freeze–Thaw Cycles and Binder Dosage on UCS

**Figure 4:**
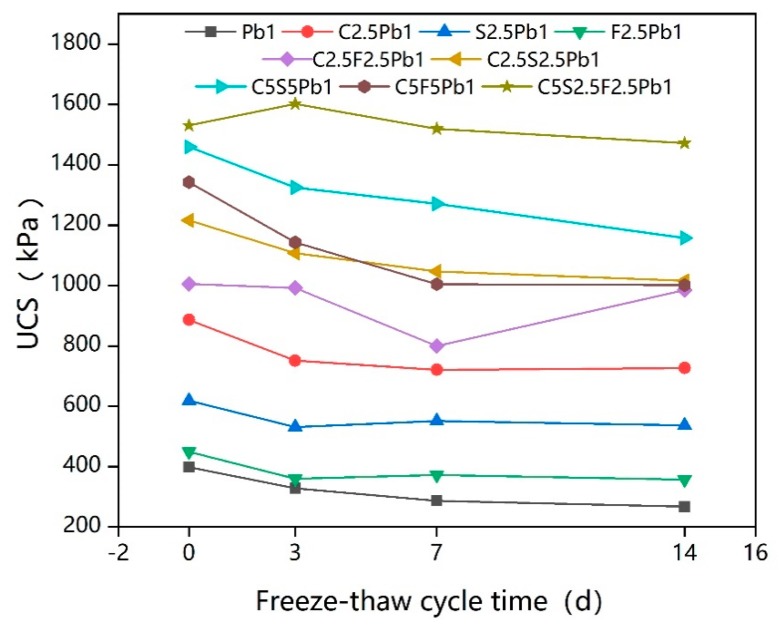
The unconfined compressive strength (UCS) values of solidified lead-contaminated soils under short-term freeze–thaw cycles.

Figure 4 shows the UCS of lead-contaminated soils treated by different types and dosages of binders under freeze–thaw cycles.

It can be clearly seen from Figure 4 that the UCS of lead-contaminated soil without binder treatment (Pb1) was the lowest, at only 398.361 kPa. However, among the samples with total binder additions of 2.5%, cement-treated lead-contaminated soil (C2.5Pb1) had the highest value of UCS, 886.388 kPa, an increase of 124%; the UCS of lead-contaminated soil after 2.5% quicklime treatment (S2.5Pb1) reached 619.055 kPa, an improvement of 55.4%, while the UCS of lead-contaminated soil (F2.5Pb1) with 2.5% fly ash reached 499.331 kPa, an increase of only 25.3%. This indicates that the addition of cement, quicklime, and fly ash all improve the UCS of lead-contaminated soil, in agreement with Kogbara [47] and Du et al. [26]. This occurs because, when cement is added to water, the main minerals in the cement, such as C_3_S (3CaO·SiO_2_), C_2_S (2CaO·SiO_2_), and C_3_A (3CaO·Al_2_O_3_), undergo a hydration reaction (as shown in Table 6), generating calcium silicate hydrate gel (C-S-H) and calcium aluminate hydrate(C-A-H), which in turn fill and close the pores of specimens and encapsulate soil particles [29,47,50]. There is also a large amount of CaO and a small amount of MgO in the cement, which undergo ion exchange, reducing the thickness of the combined water film on the surface of the soil particles and aggregating the fine-grained soil to form large particles; hardening and carbonation reactions also occur, which form insoluble crystalline materials between the soil particles, while calcium carbonate precipitation fills the pores between the soil particles and makes the soil particles denser. Furthermore, the cement hydration reactions also produce a lot of Ca(OH)_2_, which reacts with SiO_2_ and Al_2_O_3_ in a pozzolanic reaction, to produce gel products, such as hydrated calcium silicate and calcium hydrated aluminate.

Similarly to cement-cured lead-contaminated soils, the mechanism of quicklime remediation of contaminated soils has four main aspects: The ripening of quicklime, which occurs after quicklime meets water, producing Ca(OH)_2_ and giving out heat, promotes the reactions with other substances in the soils; the ion exchange actions electrolyze OH^−^ in water, which precipitates with Pb^2+^ into Pb(OH)_2_, achieving solidification and remediation of heavy metals; the carbonation reaction, in which Ca(OH)_2_ is generated after quicklime and CO_2_ in the air react to form CaCO_3_, increases the strength of specimens to a certain extent; and the pozzolanic reaction between Ca(OH)_2_ and soil minerals (such as SiO_2_ and Al_2_O_3_) produces hydrated calcium silicate, calcium hydrated aluminate, and other gel products that improve soil strength.

The UCS values of quicklime-cured lead-contaminated soils were lower than those of cement-cured lead-contaminated soils, which may be partially attributed to the differences in SiO_2_ and Al_2_O_3_ content. Although the addition of fly ash improved the UCS of specimens, such increases were significantly lower than those seen in samples with cement or quicklime. This is likely to be because the main components of fly ash are CaO, SiO_2_, and Al_2_O_3_, which are effectively hydrated only in an alkaline environment, unlike alkaline activators, such as cement and lime. Additionally, the CaO in fly ash is much lower than that in cement and quicklime (see Table 1), and thus gel-like hydration products, such as calcium silicate hydrate (CSH), and calcium aluminate hydrate (CAH), were much lower in volume. This may also explain why the UCS value of C2.5S2.5Pb1 was higher than that of C2.5F2.5Pb1 when the total amount of binder was 5%, and why the UCS value of C5S5Pb1 was higher than that of C5F5Pb1 when the total amount of binder was 10%. Due to the action of CaO and water, the pozzolanic activity of class F fly ash can be further activated to form additional gels, improving the UCS of lime-based deposits [51]. The UCS of C5S2.5F2.5Pb1 was higher than that of C5S5Pb1, supporting the studies by Dermatas and Meng [52] and Wang et al. [53]. The consensus is that the value of UCS in lead-contaminated soils increases as the total dosage of added binder increases [54,55,56].

The changes in UCS values of the nine samples based on extensions of the freeze–thaw cycle time fall roughly within two trends, as seen in Figure 4. The first shows the UCS value increasing at the beginning of the freeze–thaw cycle, and then decreasing and eventually stabilizing over the increase of the freeze–thaw cycle time. The UCS of C5S2.5F2.5Pb1 showed this trend, and the increase stage may have contributed to the action of CaO and water, activating the pozzolanic activity of the class F fly ash and forming cementitious gels [51], leading to the observed improvement in the UCS of the specimens. Alternatively, it may have attributed to the fact that the various reactions and ion exchanges may not have been completed during the solidification/stabilization process, and the reinforcement effect of the products they produced during the early freeze–thaw cycle on the lead-contaminated soil may have offset the deterioration effect of the freeze–thaw cycle [47]. However, the change in UCS values of the other eight samples showed a second trend, unlike in the first trend, there was no stage in which the UCS value increased as the freeze–thaw cycle time increased: The UCS value simply decrease with the increase in freeze–thaw cycle time before finally stabilizing. Despite this variation in trends, the conclusion is thus that the freeze–thaw cycle generally reduced the UCS value of the samples [51,57,58].

**Figure 5:**
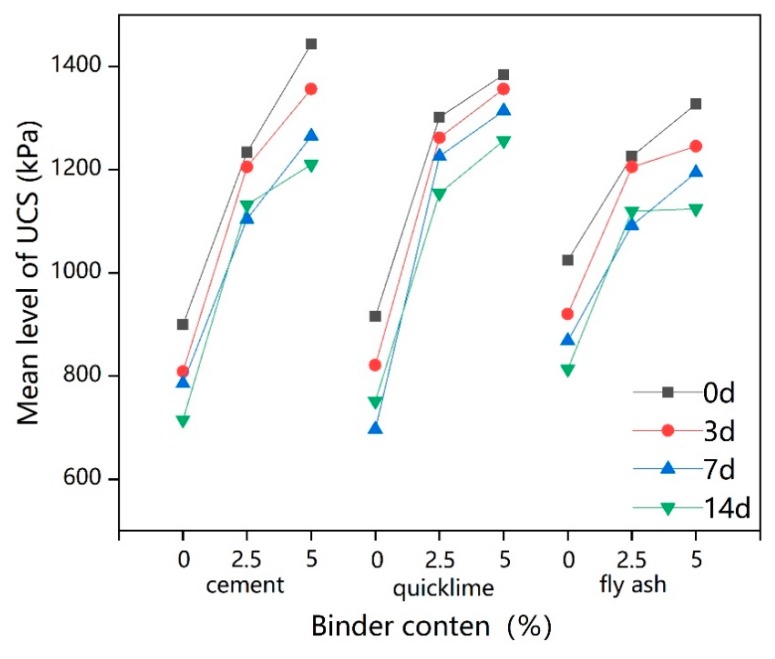
Intuitive trend chart of influencing factors for UCS.

Figure 5 shows how UCS deteriorates with increasing freeze–thaw cycles and is significantly affected by different contents levels of all three binders, especially cement and quicklime. The influence patterns on the UCS of the three binders were basically the same across the different freeze–thaw cycles. The increase in dosage of cement and quicklime from 0% to 2.5% resulted in UCS increasing significantly, while the increase of quicklime caused the largest increase in the UCS of lead-contaminated soil. When the additions increased from 2.5% to 5%, the binder that most noticeably enhanced the UCS was cement, but both cement and quicklime improved the UCS much less compared to the increased from 0% to 2.5%. This suggested that the effect of freeze–thaw cycles on the UCS of solidified lead-contaminated soil with high binder dosages is more significant. UCS also improved with the continuous increase in fly ash content, but this increase was less pronounced than that seen with the increase in cement and quicklime. When freeze–thaw cycles were relatively short (less than 3 days), the UCS values of lead-contaminated soils were most influenced by the cement content, and 5% was the best proportion of cement for improving UCS level. However, with increasing the repetition of freeze–thaw cycles, quicklime showed better performance, again being best at 5%. As a secondary factor, fly ash content may also have a positive effect, depending on the actual situation.

### 3.2. Influence of Freeze–Thaw Cycles and Binder Dosage on E_50_

**Figure 6:**
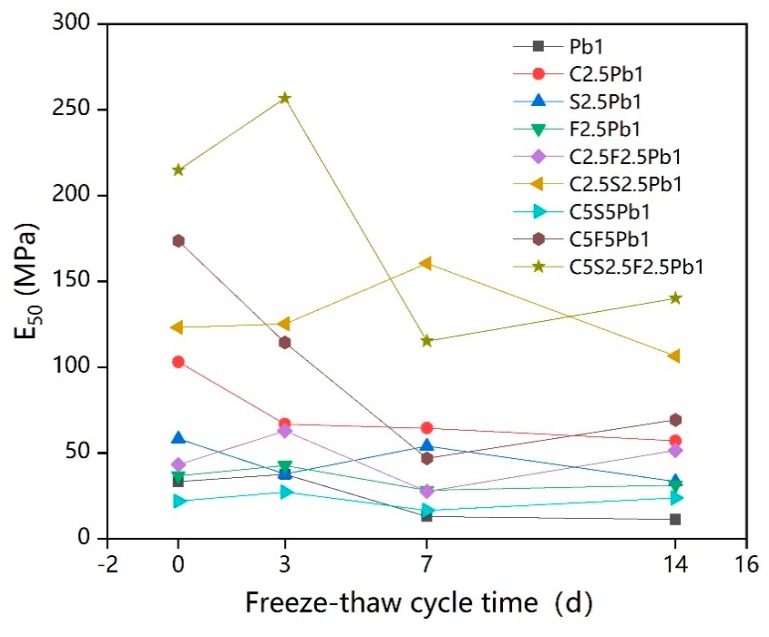
The secant modulus $\left(E_{50}\right)$ of solidified lead-contaminated soils treated under freeze–thaw cycles.

Figure 6 shows the $E_{50}$ (secant modulus) of lead-contaminated soils treated with different types and dosages of binders under various repetitions of freeze–thaw cycles.

As presented Figure 6, the addition of binders generally improved the $E_{50}$ value of lead-contaminated soils, potentially due to the cementitious gels produced by hydration and pozzolanic reactions, making the internal structures of the lead-contaminated soil more compact. For samples that had not undergone freeze–thaw cycles, it was clear that the $E_{50}$ value improved as the total dosage of binders increased. When the total binder dosage was 10%, the $E_{50}$ value of lead-contaminated soil, treated with a binder consisting of cement, quicklime, and fly ash in a 2:1:1 ratio (C5S2.5F2.5Pb1), was highest.

Based on Figure 6, the change patterns in the $E_{50}$ values of these nine samples with the increase in the freeze–thaw cycle time can be roughly divided into two types. The first one is where the $E_{50}$ value increases at the beginning of the freeze–thaw cycle, and then decreases, eventually stabilizing over the increases in the freeze–thaw cycle time, which applied to C2.5Pb1, F2.5Pb1, C2.5S2.5Pb1, C5S5Pb1, and C5S2.5F2.5Pb1. Some studies have also shown that lead is deposited as insoluble lead sulfate and carbonate on the surface of calcium silicate and aluminum silicate, forming an impervious coating that delays hydration action [59,60]. This physicochemical interaction between the hydrate and the soil might still be in progress after 56 days of curing, which would explain why the strength of solidified lead-contaminated soil would increase during early freeze–thaw cycles. However, the changing pattern in the UCS values for C5F5Pb1, C2.5Pb1, and S2.5Pb1 demonstrated the second type of pattern with no increase in the $E_{50}$ value during early freeze–thaw cycles; the $E_{50}$ values only decreased with the increase in freeze–thaw cycle time and finally stabilized. In particular, the $E_{50}$ value of C5F5Pb1 was greatly reduced in the early stages of the freeze–thaw cycle, which might be due to the large dosage of fly ash containing a greater amount of harmful substances (such as unburned carbon and heavy metals) and fine particles, which would hinder the hydration reactions of cement [53]. The main conclusion is that freeze–thaw cycles cause a reduction in the $E_{50}$ value of stabilized lead-contaminated soils, possibly due to the freeze–thaw cycles increasing the net soil volume, causing a looser soil structure and thus inevitably leading to a decrease in the elastic modulus [61]. There was a particularly big drop observed in the $E_{50}$ value of C5S2.5F2.5Pb1, which might have been caused by highly or excessively cemented soil due to abundant cementitious gels being produced from the solidification/stabilization process [62]. As early as in 1998, data from Viklander [63] prove that in very dense soils, porosity might increase due to freezing and thawing, where soil particles did not completely return to their original location after each stage, leading to an increase in the net soil volume and, and a looser soil structure than before freezing. And it is clear from Figure 6 that the solidified lead-contaminated soils with higher dosages of binders, such as C5S2.5Pb1 and C5F5Pb1, showed larger changes with the increase in freeze–thaw cycle time, suggesting that the deterioration effect of freeze–thaw cycles is more evident on solidified contaminated soils with larger dosages of added binders.

**Figure 7:**
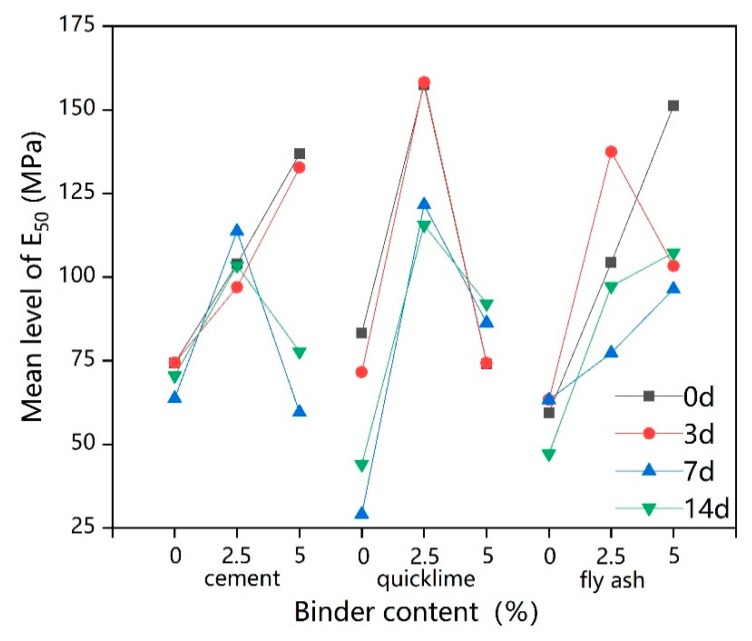
Intuitive trend chart of influencing factors for $E_{50}$.

The intuitive trend chart of influencing factors for $E_{50}$ (Figure 7) shows that the $E_{50}$ value increased approximately linearly with increases in the cement content when freeze–thaw cycle time was relatively short (0 days, 3 days), but that, with increasing time of freeze–thaw cycles (7 days, 14 days), it reached a maximum when the cement content was 2.5%. Under all conditions, $E_{50}$ was noticeably increased by increases in quicklime content from 0% to 2.5%, decreasing sharply as the content increased from 2.5% to 5%. As seen in Figure 7, at 7 days and 14 days, when the dosages of cement and quicklime were both 2.5%, their curves reached their highest points [64]. However, for the same longer freeze–thaw cycles (7 days and 14 days), the $E_{50}$ value reached its maximum value when the dosage of fly ash was 5%. The reason might be that the degree of hydration of fly ash was not as good as that of cement and quicklime, making it harder to produce excessive cementation in the solidified lead-contaminated soils and causing strength loss [29,62]. This is consistent with the phenomena (shown in Figure 6) where the $E_{50}$ value of C5S2.5F2.5Pb1 was higher than that of C5S5Pb1. $E_{50}$ could thus be improved by using a 2.5% addition of quicklime. When considering the effect of long-term freeze–thaw cycles, Figure 7 also suggests that the value of $E_{50}$ was best when the addition dosages of cement and quicklime were both 2.5%, and the addition dosage of fly ash was 5%.

### 3.3. Influence of Freeze–Thaw Cycles and Binder Dosage on Shear Index

**Figure 8:**
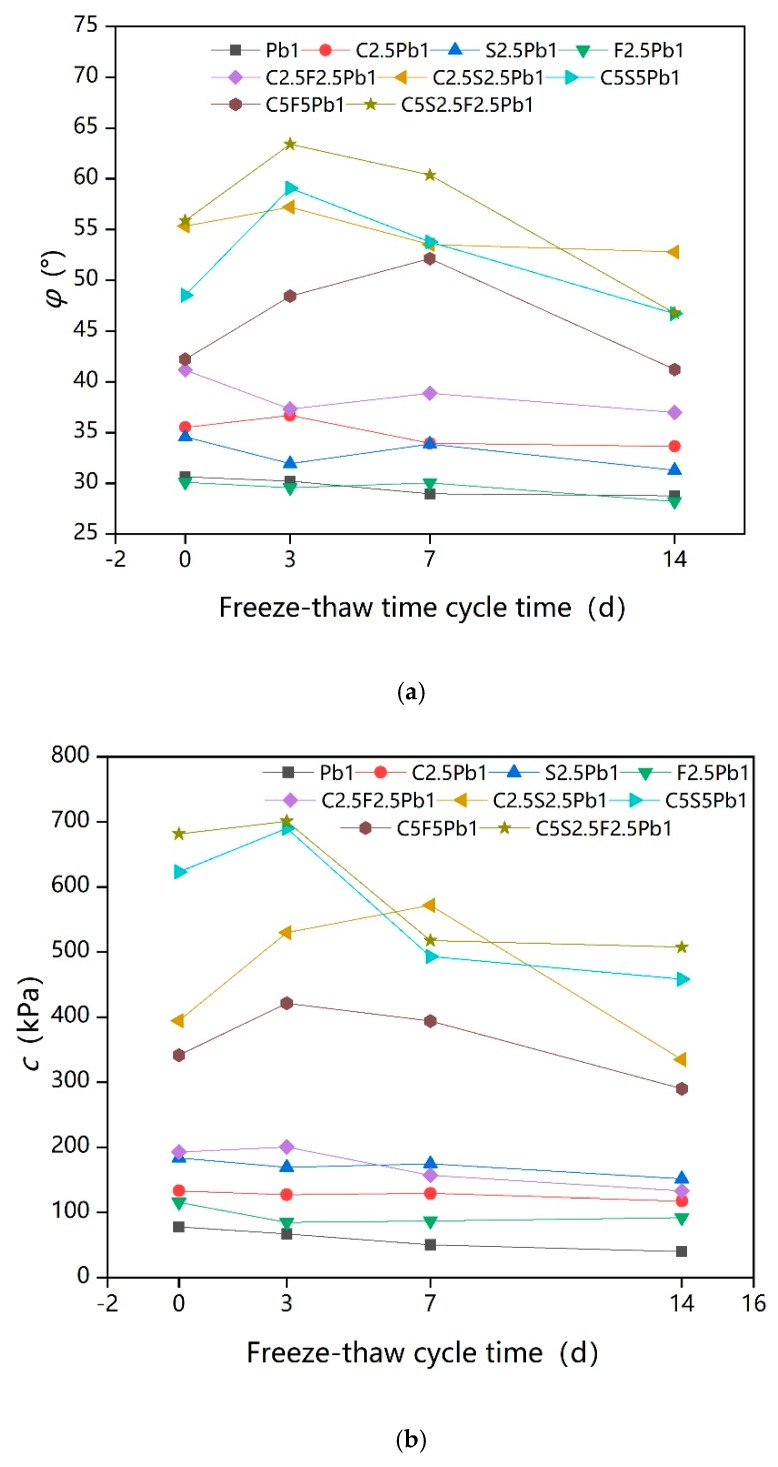
The shear index of solidified lead-contaminated soils under freeze–thaw cycles: (**a**) The internal friction angle (φ) of solidified lead-contaminated soils under freeze–thaw cycles; (**b**) The cohesion (c) of solidified lead-contaminated soils under freeze–thaw cycles.

Figure 8 shows the shear index of lead-contaminated soils treated with different types and dosages of binders under freeze–thaw cycles.

Values for unfrozen stabilized lead-contaminated soils are also included in Figure 8, showing that there was almost no difference in the internal friction angle φ of the solidified lead-contaminated soil with 2.5% fly ash addition (F2.5Pb1) from that of untreated the lead-contaminated soil, while the cohesion, c, showed only a small increase compared to the lead-contaminated soil. This shows that a simple addition of 2.5% fly ash dose not obviously improve the φ and c values of lead-contaminated soils (Pb1), in agreement with Zhao and Yang [65]. Although the addition of 2.5% cement/quicklime has been shown to increase the φ and c of lead-contaminated soil [66,67], these increases are not as good as those driven by the use of multiple binders [65]. Moreover, previous studies have shown that, under the same dosage of binder, the shear index of clay stabilized with quicklime alone is not as good as the shear index of clay stabilized with fly ash-quicklime [68,69], which could help explain why the C5S2.5F2.5Pb1 had the largest φ and c values, about 1.5 times and 7 times of those of Pb1, respectively. In general, when the total dosage of binder was increased to 5% then 10%, the φ and the c values were significantly improved.

Figure 8 also presents that as freeze–thaw cycle time increases, the shear index of the nine samples showed two trends overall. For the solidified lead-contaminated soil with a lower binder or absent dosages (0% and 2.5%), the shear index decreased slightly with the increase in freeze–thaw cycle time; however, overall, any change was very small. This implies that freeze–thaw cycles have little effect on the shear index of lead-contaminated soil and of repaired lead-contaminated soils with small dosages of binder. However, for solidified lead-contaminated soil with higher binder dosage (5% and 10%), the value of the shear index increased during early freeze–thaw cycles and then decreased as the effect of freeze–thaw cycles were extended. The reason for the increase in shear index in the early stage is likely to be that the solidification/stabilization process was still ongoing after 56-days [67,69] and that the cementation of the contaminated soils’ structure caused by the products of the solidification/stabilization process in the early stage of the freeze–thaw action was sufficient to offset some deterioration caused by the freeze–thaw cycles. However, intensification of the freeze–thaw cycles alongside an increase in hydration products causes the soil to become over cemented [62] means that the subsequent shear strength is significantly reduced. Overall, the freeze–thaw cycle is not favorable for the shear strength of solidified contaminated soil [70].

**Figure 9:**
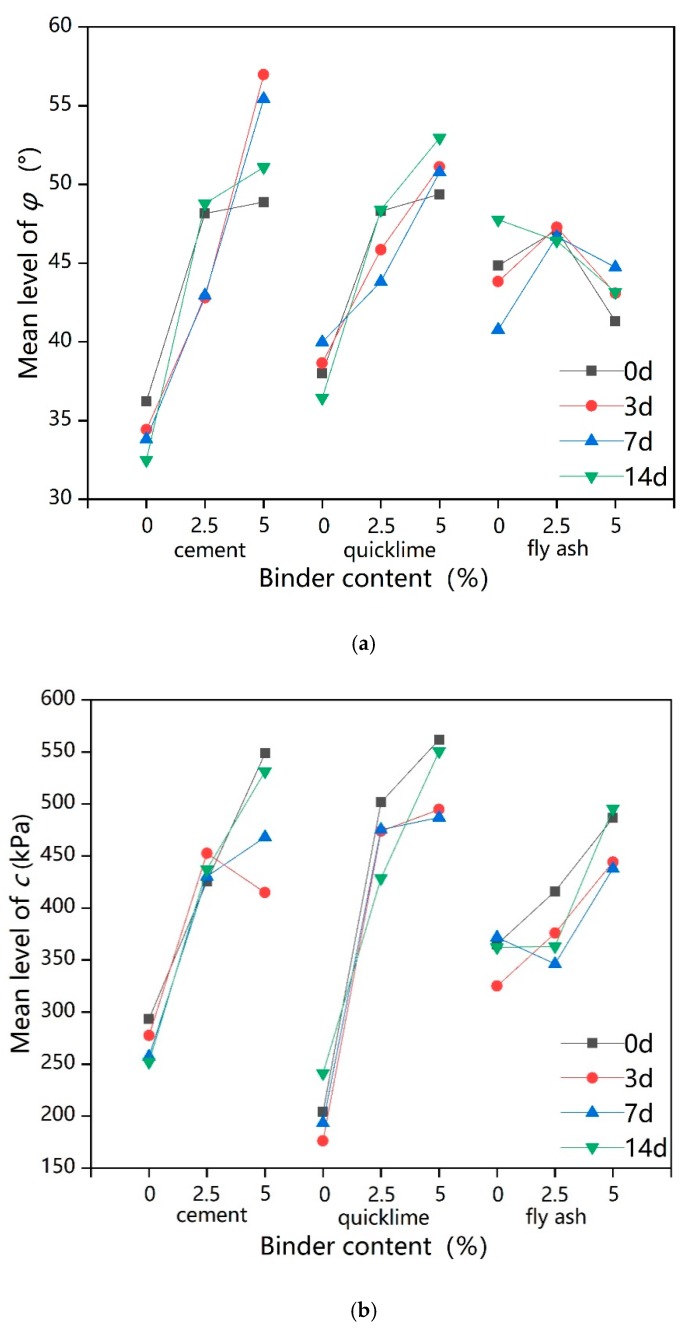
Intuitive trend chart of influencing factors for the shear index: (**a**) Intuitive trend chart of influencing factors for φ; (**b**) Intuitive trend chart of influencing factors for c.

Figure 9a shows that the φ was affected to varying degrees by the dosages of cement, quicklime, and fly ash. When the freeze–thaw cycle time was 0 days or 14 days, the influencing curves of cement and quicklime were similar; as the dosages increase from 2.5% to 5%, the increase in φ was less than where the dosages increase from 0% to 2.5%, and the trend for φ value was approximately linear as the dosages of cement and quicklime increase when freeze–thaw cycle time was 3 days and 7 days. The influencing curve of fly ash demonstrates a highest point at a 2.5% binder dosage under all four freeze–thaw cycles; the optimum content of fly ash for φ was thus 2.5%. Considering the long-term freeze–thaw effect, the addition of 5% cement and 5% quicklime are better for improving φ values.

Figure 9b also shows that c was noticeably improved by increasing the content of cement and quicklime, except when the freeze–thaw cycle time was 3 days or 7 days. Further, the improvement from cement or quicklime content increasing from 2.5% to 5% was minimal compared to the increase seen when the addition was increased from 0% to 2.5%, especially with regard to quicklime. The results indicate that the c of lead-contaminated soils can be immensely improved by adopting a 5% cement, 5% quicklime, or 5% fly ash addition.

### 3.4. Influence of Freeze–Thaw Cycles and Binder Dosage on K

**Figure 10:**
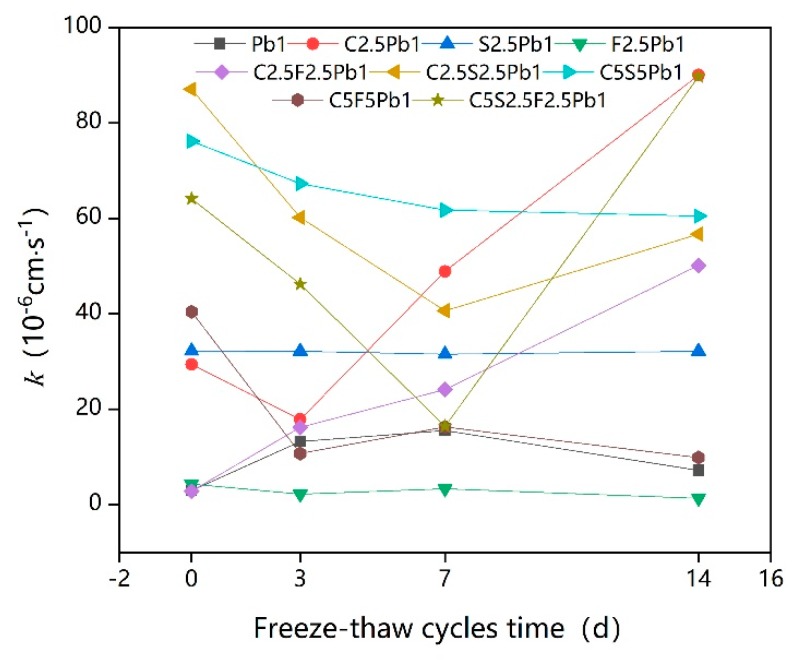
The permeability coefficient (k) of solidified lead-contaminated soils under freeze–thaw cycles.

Figure 10 shows the k (permeability coefficient) of lead-contaminated soils treated with different types and dosages of binders under various freeze–thaw cycles.

The permeability coefficient k of samples without freeze–thaw cycles increased with the increase in the dosage of binder, in contrast to the expected decrease in k with the increase in the dosage of binder. A similar result was reported by Kogbara and Al-Tabbaa [71], who showed a significant increase in permeability with increasing lime-slag mixed binder dosage. Elrawi and Awad [72] further showed that the k of lime-stable sandy silty clay increased with increasing lime dosage, which could explain why the k value of C5F5Pb1 was smaller than that of C5S5Pb1 and the k value of C2.5F2.5Pb1 was smaller than that of C2.5S2.5Pb1. Quigley [73] indicated that the adsorption of Pb by soil particles and the adsorption of both by cement occurs during solidification/stabilization, making the distance between the soil particle layers smaller, resulting in further aggregation of soil particles; as a result, the seepage distance increases and the k of the solidified/stabilized lead-contaminated soils increases intensely. As the gel-like hydration products of fly ash are minimal compared to those of cement and quicklime, the addition of fly ash did not significantly increase the k of lead-contaminated soils. Nevertheless, the k value generally increased with an increase in the binder dosage [10].

The broken line in Figure 10 highlights the four types of changes in k seen with the extension of the freeze–thaw cycle time. The first is where the value of k was not greatly affected by the freeze–thaw cycle, as in F2.5Pb1 and S2.5Pb1. The second one is where the k value continuously decreased with the increase in the freeze–thaw cycle time, as in C5S5Pb1 and C5F5Pb1. As mentioned earlier, an excessive binder dosage can result in too many hydration products occurring, which leads to excessive cementation and compaction of solidified contaminated soil, widening the seepage channels and thus eventually increasing k [62,73]. The freezing and thawing cycle then destroys the big aggregates, though the frozen and thawed soil particles might not completely return to their original positions, causing excessive cementation and making the compact soil become loose and split into small aggregates, causing seepage channels to be blocked and making k decrease [61,63,74].

The third pattern is where the value of k. decreased during early the freeze–thaw cycle, and then increased as the duration of the freeze–thaw cycle increased, as seen in C2.5S2.5Pb1, C5S2.5F2.5Pb1, and C2.5Pb1. The reason for the decline is as seen in the second trend with the reason for the subsequent increase being that the freeze–thaw cycle relieved the excessive cementation of the soil before continuing to destroy the original structure of the soil particles, causing the formation of polygonal shrinkage cracks and a reduction in the volume of fine powder in the pores of the coarse fraction, which eventually appeared as an increase in k [74,75]. The reason why the k values of C5S5Pb1 and C5F5Pb1 did not appear to rise was that their binder contents were higher, and thus, their degrees of cementation were higher. It can thus be predicted that if the freeze–thaw cycle was continued, the k value would increase. The final pattern is where the k values of Pb1 and C2.5F2.5Pb1 increased continuously with the increase in the freeze–thaw cycle time, suggesting that the freeze–thaw cycle destroyed the cementite structure of the solidified contaminated soil, making the over-solidified contaminated soil structure looser as, the large cementite structure split into smaller aggregates. This makes the k value smaller, and as long as the freeze–thaw cycle continues, the structure of the solidified contaminated soil continues to be damaged, causing large fissures and reducing the levels of fine particles in the larger pores, leading to further increases in permeability [76,77].

**Figure 11:**
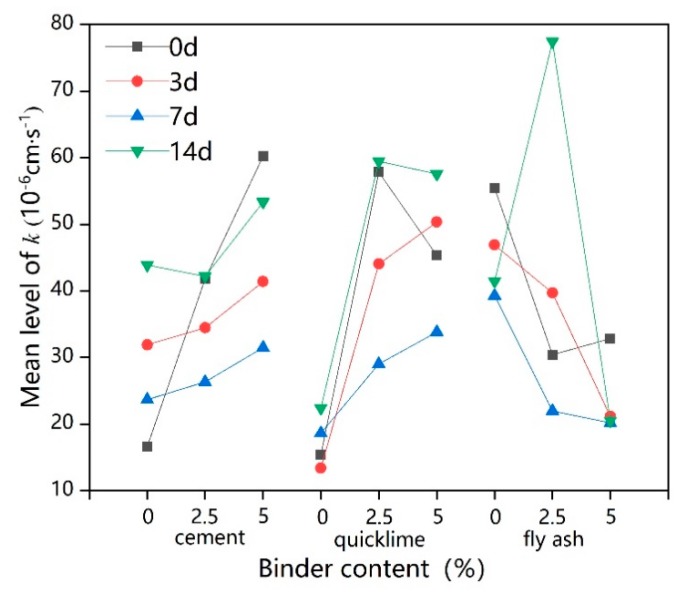
Intuitive trend chart of influencing factors for k.

Intuitively from Figure 11, the k value continued to increase as the dosages of cement or quicklime increased, while it decreased rapidly with increases in fly ash dosage [78]. A better level of k may thus be achieved by adopting a 5% fly ash, while the dosage of cement/quicklime should be reduced as far as possible.

## 4. Conclusions

In this work, unconfined compressive strength tests, direct shear tests, and permeability tests were performed to investigate the values of UCS, $E_{50}$, φ, c, and k in compound solidified/stabilized lead-contaminated soils under various freeze–thaw cycles and different binder dosages (0%, 2.5%, 5%). By adding an intuitive trend graph method to examine the influencing factors, this article further explored the issue of the best binder dosage for improving the properties of solidified/stabilized lead-contaminated soil under the action of short-term freeze–thaw cycles. The main conclusions are as follows:

Generally, the unconfined compressive strength (UCS), the secant modulus ($E_{50}$), and the shear index (φ and c) increased with increased dosages of binders, which confirmed the remediation effect of all tested binders on lead-contaminated soil.

The permeability coefficient (k) increased with the increase in binder to a certain extent, which suggesting that it is incorrect to think that the higher the dosage of binder, the better the curing effect; there appears to be a critical dosage of added binder for contaminated soils with certain heavy metal content, and excessive cementation of contaminated soil caused by adding too much binder can result in loss of strength and an increase in permeability.

The freeze–thaw cycle had an adverse effect on the engineering characteristics of solidified lead-contaminated soils, shown by decreases in UCS, ${\;E}_{50}$, φ, and c values and k value increases with increases in freeze–thaw cycle time. For the solidified lead-contaminated soils with higher binder dosages, this degradation effect was even more obvious.

However, as the solidification/stabilization process was still in process, further reinforcement of the contaminated soil by the products of hydration reactions and adsorption sedimentation appeared to offset the deterioration caused by the freeze–thaw cycles, so that degradation was not severe in the early stage of the freeze–thaw cycle, and UCS, $E_{50}$, φ, and c values appeared to increase.

As excessive solidification and over cementation of the solidified contaminated soil was caused by an excess of binder, where the freeze–thaw cycle time was relatively short, the damage to the structure by the freeze–thaw cycle actually caused the solidified contaminated soil to cease being over cemented; similarly, the k value consistently decreased with the increase of freeze–thaw cycle time.

Under the long term action of freeze–thaw cycles, adding 5% cement and 5% quicklime could show the better results for the UCS and shear index in lead-contaminated soils (1% lead), while the value of $E_{50}$ was best when the addition dosage of fly ash was 5%. However, to avoid excessive cementation of solidified contaminated soil, 2.5% fly ash and 2.5% cement/quicklime were sufficient to improve the permeability and $E_{50}$ of lead-contaminated soils to an excellent condition, respectively.

## Figures and Tables

**Table 1:** Basic physical properties of undisturbed soil samples.

Physical Property Index	Liquid Limit/%	Plastic Limit/%	$\mathrm{Plasticity}\;\mathrm{Index}\;\left(I_{p}\right)$	Optimum Moisture Content/%	$\mathrm{Maximum}\;\mathrm{Dry}\;\mathrm{Density}/g/cm^{3}$
soil sample	28.6	16.7	11.9	13.65	1.842

**Table 2:** Main chemical components of tested soil (expressed as oxides).

Chemical Components	$SiO_{2}$	$Al_{2}O_{3}$	$Fe_{2}O_{3}$	CaO	MgO	$SO_{3}$	$K_{2}O$	$TiO_{2}$	$Na_{2}O$	PbO
Contents (%)	65.24	17.27	5.14	3.32	3.46	0.04	3.47	0.74	0.83	ND

Note: ND means not detected.

**Table 3:** Quality index for $\mathrm{Pb}{\left({\mathrm{NO}}_{3}\right)}_{2}$.

Molecular Formula	$Pb{\left(NO_{3}\right)}_{2}$
Molecular Weight	331.21
Content	≥99.0%
Cu	≤0.0005%
Fe	≤0.001%
Cl	≤0.001%
Hydrocarbon that does not precipitate (based on sulfate)	≤0.05%
Clarity experiment	qualified
pH value (50 g/L,25 °C)	≤0.003%

**Table 4:** Chemical composition of binders (expressed as oxides).

Chemical Components		CaO	MgO	$SiO_{2}$	$Al_{2}O_{3}$	$Fe_{2}O_{3}$	$SO_{3}$	$TiO_{2}$	$Na_{2}O$
**Content (%)**	**Cement**	49.18	1.62	26.01	10.67	2.83	3.76	0.51	0.13
	**Quicklime**	84.23	4.32	3.10	3.10	0.29	ND	ND	ND
	**Fly Ash**	5.73	3.68	39.65	21.42	9.17	ND	ND	2.03

Note: ND means not detected.

**Table 5:** Experimental scheme.

Freeze–Thaw Cycles Time	Freeze–Thaw Strength	Test Program	Specimen Number
0,3,7,14	−10 °C~20 °C	UCS test Direct shear test Permeability test	Pb1(for comparison) C2.5Pb1; S2.5Pb1; F2.5Pb1 C2.5F2.5Pb1; C2.5S2.5Pb1 C5S5Pb1; C5S2.5F2.5Pb1; C5F5Pb1

**Table 6:** Hydrolysis hydration of cement.

Mineral Name	Chemical Composition	Hydration Reaction Process
C_3_S	3CaO·SiO_2_	2$\left(3\mathrm{CaO}\cdot {\mathrm{SiO}}_{2}\right)$ + 6$H_{2}O\to 3\mathrm{CaO}\cdot 2{\mathrm{SiO}}_{2}\cdot 3H_{2}O$ + $3\mathrm{Ca}{\left(\mathrm{OH}\right)}_{2}$
C_2_S	2CaO·SiO_2_	2$\left(2\mathrm{CaO}\cdot {\mathrm{SiO}}_{2}\right)$ + 6$H_{2}O\to 3\mathrm{CaO}\cdot 2{\mathrm{SiO}}_{2}\cdot 3H_{2}O$ + $3\mathrm{Ca}{\left(\mathrm{OH}\right)}_{2}$
C_3_A	3CaO·Al_2_O_3_	$\mathrm{CaO}\cdot {\mathrm{Al}}_{2}O_{3}+6H_{2}O\to 3\mathrm{CaO}\cdot {\mathrm{Al}}_{2}O_{3}\cdot 6H_{2}O$
C_4_AF	4CaO·Al_2_O_3_·Fe_2_O_3_	$4\mathrm{CaO}\cdot {\mathrm{Al}}_{2}O_{3}\cdot {\mathrm{Fe}}_{2}O_{3}+2\mathrm{Ca}{\left(\mathrm{OH}\right)}_{2}+10H_{2}O\to 3\mathrm{CaO}\cdot {\mathrm{Al}}_{2}O_{3}\cdot 6H_{2}O+3\mathrm{CaO}\cdot {\mathrm{Fe}}_{2}O_{3}\cdot 6H_{2}O$
